# Real-world effectiveness and safety of ibrutinib in relapsed/refractory mantle cell lymphoma in Japan: post-marketing surveillance

**DOI:** 10.1007/s12185-023-03687-8

**Published:** 2024-01-09

**Authors:** Dai Maruyama, Ai Omi, Fumi Nomura, Tokiko Touma, Yukiko Noguchi, Kyoko Takebe, Koji Izutsu

**Affiliations:** 1 Department of Hematology Oncology, Cancer Institute Hospital, Japanese Foundation for Cancer Research, Tokyo, Japan; 2 Medical Affairs Division, Janssen Pharmaceuticals K.K., 3-5-2 Nishi-kanda, Chiyoda-ku, Tokyo, 101-0065 Japan; 3 Japan Safety and Surveillance Division, Janssen Pharmaceuticals K.K., Tokyo, Japan; 4 Statistics and Decision Sciences Japan, Janssen Pharmaceuticals K.K., Tokyo, Japan; 5 Department of Hematology, National Cancer Center Hospital, Tokyo, Japan

**Keywords:** Ibrutinib, Mantle cell lymphoma, Post-market surveillance, Real-world evidence, Safety

## Abstract

Efficacy and safety data for ibrutinib in Japanese patients with relapsed/refractory (R/R) mantle cell lymphoma (MCL) were limited at the time of its approval in Japan. All-case post-marketing surveillance was conducted in Japanese R/R MCL patients who began ibrutinib treatment between December 2016 and December 2017, and patients were followed until 30 June 2020. In the effectiveness analysis set (*n* = 202), the overall response rate was 59.9%, 52-week progression-free survival was 47.5%, and overall survival was 69.3%. Safety was assessed in 248 patients (median age 74.0 years). When ibrutinib treatment was started, patients had received a median of three prior lines of therapy. The overall incidence of adverse events (AE) was 74.6%, and AE frequency and severity grade distribution were similar between patients with 1 versus more than 1 prior line of therapy. The most common AE was platelet count decreased (all grades; 10.4%), similarly to previous observations in patients with R/R chronic lymphocytic leukemia/small lymphocytic lymphoma. Five patients (2.0%) developed atrial fibrillation. The effectiveness and safety of ibrutinib were consistent with its known profile at approval in Japan. These results suggest that ibrutinib is effective and safe in Japanese R/R MCL patients in routine clinical practice.

**Supplementary Information:**

The online version contains supplementary material available at 10.1007/s12185-023-03687-8.

## Introduction

Mantle cell lymphoma (MCL) is a relatively rare form of B-cell non-Hodgkin lymphoma (NHL) [1], comprising approximately 3.0% of all NHL cases in Japan [2].

A common feature of MCL is cyclin D1 overexpression, resulting from the translocation of t(11; 14) (q13; q32) [3]. While MCL can affect patients at any age, it is more common in elderly people, with 75.0% of MCL patients in Japan aged over 65 years [4]. Although a small proportion of patients with MCL have an indolent or smoldering form of the disease at diagnosis and can, therefore, defer treatment, most MCL patients (70.0–80.0%) present with symptomatic disease that requires immediate treatment [5].

For Ann Arbor stage III or IV MCL, first-line preferred treatment is dependent on age and eligibility for treatment with high-dose chemotherapy/autologous hematopoietic stem cell transplantation (HDC/AHSCT). In patients who are < 65 years or eligible for HDC/AHSCT, Japanese guidelines recommend cytarabine-containing intensive induction regimens followed by transplantation [6], which have been shown to be effective [7]. In those who are > 65 years or not eligible for HDC/AHSCT, rituximab plus CHOP (cyclophosphamide, doxorubicin, vincristine, and prednisone) followed by rituximab maintenance, VR-CAP (rituximab, cyclophosphamide, doxorubicin, prednisone plus bortezomib), or rituximab plus bendamustine are recommended in the Japanese guidelines [6].

Ibrutinib is a first-in-class, once-daily oral Bruton's tyrosine kinase inhibitor [8] that is approved in Japan for the treatment of B-cell malignancies, including chronic lymphocytic leukemia/small lymphocytic lymphoma (CLL/SLL) and MCL, as well as for chronic graft versus host disease [9]. The efficacy and safety of ibrutinib in Japanese patients with R/R MCL were confirmed in a domestic phase II study [10, 11], as well as in international studies [12–15]. However, the Japanese phase II study included only 16 patients; thus, data for ibrutinib in Japanese patients are limited.

The current article describes post-marketing surveillance (PMS) that investigated the effectiveness and safety of ibrutinib treatment for up to 52 weeks in Japanese patients with R/R MCL in routine clinical practice.

## Materials and methods

### Study design, patients, and data collection

This observational, multicenter PMS was conducted at 180 centers in Japan between December 2016 and 30 June 2020 (UMIN-CTR Clinical Trials Register ID: UMIN000028130).

All patients with R/R MCL (relapse or refractoriness determined by the treating physician) who began treatment with ibrutinib in the first year after ibrutinib approval (December 2016 to 31 December 2017) were included and followed up for 52 weeks after ibrutinib initiation or until the date of ibrutinib discontinuation or patient loss to follow-up.

Anonymized patient data were collected via a survey form utilizing an electronic data capture system. Patient data were recorded in electronic case report forms (eCRFs) at baseline, and at 12 and 52 weeks after the first ibrutinib dose.

This surveillance was conducted in compliance with Japanese Good Post-Marketing Study Practice regulations. The protocol was reviewed and approved by the Pharmaceuticals and Medical Devices Agency of Japan. As this was a mandatory PMS assessing the use of ibrutinib in an approved indication and anonymized data were collected from clinical settings, informed consent from the patients was not required for the collection and use of their data.

### Assessments

In order to evaluate the types of patients receiving ibrutinib, the data collected in the eCRF at baseline included demographics (sex, age, height, body weight, and pregnancy or breastfeeding status); treatment setting (in- or outpatient); disease-related characteristics (time of disease onset and recurrence/s, t(11; 14) translocation status, Ann Arbor classification, presence of B symptoms or bulky disease [lesions ≥ 10 cm], and Eastern Cooperative Oncology Group performance status); medical history and concurrent diseases (particularly infectious complications; serious bone marrow failure; arrhythmias; renal impairment; hepatic function disorder; and history of hemorrhage, hyperlipidemia, severe accidents or injuries, or psychoneurotic disorders); other treatments, including blood transfusions, stem cell transplants, and concomitant medications, including supportive/prophylactic therapies; prior treatments for MCL; ibrutinib dose; and laboratory test results.

In order to evaluate how ibrutinib is used in clinical practice in Japan, the following data were recorded in the eCRF during follow-up: ibrutinib treatment (dose changes/interruptions/discontinuation, treatment duration), changes to other treatment parameters, and laboratory tests. At the completion of the observation period, the following information was recorded: ibrutinib administration status, and confirmation of survival or death.

Effectiveness was assessed using response evaluations recorded in the eCRFs, with timing of evaluation at the discretion of the treating physician. Response was evaluated based on the revised response criteria for malignant lymphoma [16]. Best overall response (including complete response [CR], partial response [PR], stable disease, or progressive disease [PD]) was evaluated during the period from baseline (ibrutinib initiation) to Week 52 (or date of last ibrutinib dose). Clinical response was defined as CR or PR. Progression-free survival (PFS) was defined post hoc as the time from the start of ibrutinib treatment to the date of disease progression or death from any cause, whichever occurred first. Study patients without documented disease progression or lost to follow-up for reasons other than death were censored at the date of last confirmation of survival by the physician. Overall survival (OS) was measured from ibrutinib initiation until the date of death before the end of the observation period. When no event was recorded, the patient’s data were censored at 52 weeks. A post hoc analysis of patients who continued treatment with ibrutinib after being assessed as having PD at some point during the observation period was also conducted.

Safety was evaluated by collecting data on the frequency and severity of adverse events (AEs), including evaluation of AEs that required dose modification or treatment discontinuation. AEs were coded in accordance with the Medical Dictionary for Regulatory Activities (MedDRA) version 23.0, and graded according to the National Cancer Institute Common Terminology criteria for Adverse Events, version 4.0. The time to onset, causal relationship with ibrutinib, and outcome of AEs were also recorded.

The following were defined as AEs of special interest: hemorrhage, bone marrow depression, infections, arrhythmia, hypersensitivity, tumor lysis syndrome, eye disorders, hepatic failure/hepatic function disorder, interstitial lung disease, leukocyte disorders, oculomucocutaneous syndrome (Stevens-Johnson syndrome), and secondary cancers. Secondary cancers were defined based on MedDRA systems organ class (SOC) terminology ‘Neoplasms benign, malignant, and unspecified [including cysts and polyps]’, and thus included exacerbation and recurrence of MCL (in accordance with Japanese regulatory requirements), although any AEs with MedDRA lower level and preferred terms containing ‘benign’ were excluded.

### Statistical analysis

A sample size of 200 patients was planned for this surveillance, based on the incidence of AEs of special interest reported in a Japanese phase II clinical study in patients with R/R MCL (Study PCI-32765MCL2002) [10, 11], and to enable comparison with a similar PMS in 200 patients with R/R CLL/SLL [17].

The safety analysis set was defined as all patients who had an eCRF collected, had received at least one confirmed dose of ibrutinib, and had not violated the PMS protocol or received ibrutinib prior to entering the PMS. The safety analysis set was used for baseline, demographics, exposure, and safety analyses. The effectiveness analysis set was defined as all patients in the safety analysis set who had an effectiveness evaluation available. No imputation was made for missing data.

Data were summarized using descriptive statistics, including mean, standard deviation (SD), and median for continuous variables, and proportions for discrete variables. The overall response rate (ORR; the proportion of patients with CR or PR) in the effectiveness analysis set was calculated. The Kaplan–Meier method was used to evaluate median PFS, and PFS and OS rates at 52 weeks. The Fisher’s exact test was employed for 2-level factorials, and the 2 × *n* chi-squared test was used for 3- or more level factorials to compare proportions. Differences were considered significant at *P* < 0.05 without multiplicity adjustment.

All statistical analyses were performed using SAS^®^ version 9.4 statistical software (SAS Institute, Cary, NC).

## Results

### Patient disposition and demographics

**Fig. 1:**
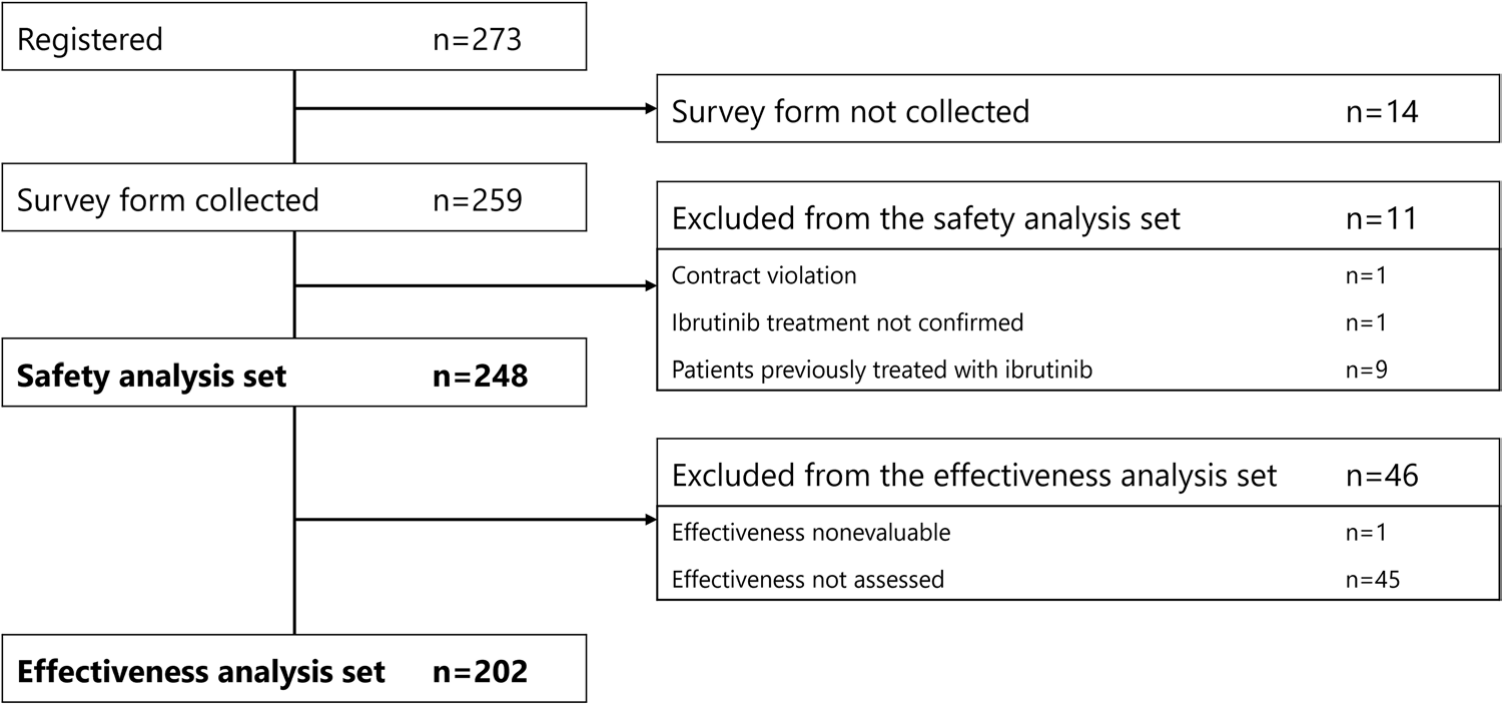
Post-marketing surveillance populations

Overall, 273 patients were registered for the PMS, and eCRFs were collected from 259. The safety analysis set included 248 patients and the effectiveness analysis set included 202 patients (Fig. 1).

Patients in the safety analysis set were aged 26.0–91.0 (median 74.0) years (Table 1), with most (69.4%) aged ≥ 70.0 years. The majority of patients were male (77.8%). The indication for ibrutinib was refractory MCL in 73 patients (29.4%) and relapsed MCL in 175 (70.6%), and most patients (*n* = 173 [69.8%]) had Ann Arbor stage IV disease. In total, 117 patients (47.2%) had t(11; 14) translocation, 185 (74.6%) had expression of cyclin D1 identified, and 31 (12.5%) had bulky disease. MCL was confirmed by genetic testing in 199 patients (80.2%). Patients had received a median (range) of 3.0 (1.0–12.0) prior lines of therapy. The most common prior therapy was combination therapy with rituximab + bendamustine (Supplementary Table S1, Electronic Supplementary Materials).

**Table 1:** Demographic and baseline clinical characteristics (safety analysis set)^a^

	Safety analysis set (*N* = 248)
Age, years, median (range)	74.0 (26.0–91.0)
Age category, *n* (%)	
< 70 years	76 (30.6)
≥ 70 years	172 (69.4)
Sex, male, * n* (%)	193 (77.8)
Indication for ibrutinib, * n* (%)	
Refractory MCL	73 (29.4)
Relapsed MCL	175 (70.6)
t(11;14) translocation, * n* (%)	
Yes	117 (47.2)
No	29 (11.7)
Unknown^b^	102 (41.1)
Expression of cyclin D1, * n* (%)	
Yes	185 (74.6)
No	10 (4.0)
Unknown^b^	53 (21.4)
Ann Arbor stage, * n* (%)	
I	11 (4.4)
II	24 (9.7)
III	34 (13.7)
IV	173 (69.8)
Unknown	6 (2.4)
ECOG PS, * n* (%)	
0	85 (34.3)
1–2	141 (56.9)
3–4	20 (8.1)
Unknown	2 (0.8)
Number of prior therapies, median (range)	3.0 (1.0–12.0)
Number of prior therapies, * n* (%)	
1	43 (17.3)
2	51 (20.6)
3	56 (22.6)
≥ 4	98 (39.5)
Bulky disease (≥ 10 cm), * n* (%)	
Yes	31 (12.5)
No	216 (87.1)
Unknown	1 (0.4)
Time from diagnosis, years, median (range)	3.7 (0.1–25.3)

*ECOG PS* Eastern Cooperative Oncology Group Performance Status, *MCL* mantle cell lymphoma, *n* number of patients

^a^Baseline was the time point at which treatment with ibrutinib was initiated

^b^Includes patients who were tested but for whom there were no test results, and patients who were not tested

### Ibrutinib treatment

The median (range) follow-up duration was 6.1 (0.1–13.1) months. At the end of follow-up, 98 patients were still receiving ibrutinib and 150 (60.5%) had discontinued treatment, mostly because of insufficient effect (*n* = 56 [22.6%]), AEs (*n* = 39 [15.7%]), or death (*n* = 28 [11.3%]; Table 2). Eighty-seven patients (35.1%) required a dose reduction or a temporary interruption, mainly because of AEs (*n* = 78 [31.5%]; Table 2).

**Table 2:** Treatment details and patient disposition (safety analysis set)

Patient disposition	Safety analysis set (*N* = 248)
Median (range) follow-up, months	6.1 (0.1─13.1)
Dose reduction or interruption, * n* (%)	87 (35.1)
AE^a^	78 (31.5)
Other^a^	19 (7.7)
Unknown	1 (0.4)
Discontinuation, *n* (%)	150 (60.5)
Insufficient effect^b^	56 (22.6)
AE except for death^b^	39 (15.7)
Death^b^	28 (11.3)
Other	27 (10.9)

*AE* adverse event, *n* number of patient

^a^Allowing for overlapped patients

^b^Including patients with progressive disease who were analyzed in the effectiveness analysis set

The mean ± SD daily dose of ibrutinib during the observation period was 456.2 ± 129.5 mg.

### Effectiveness

In the effectiveness analysis set (*n* = 202), the ORR was 59.9%, and the median time to best response was 77.0 days. Fifty-six patients (27.7%) achieved CR, 65 (32.2%) had PR, 34 (16.8%) had stable disease, and 46 (22.8%) had PD. Data from one patient in the effectiveness analysis set was classified as “missing/unlisted", even though a best response was available for this patient (PR). For this patient, the end date of the observation period was incorrectly entered and, as such, we were unable to determine if this effect measurement was performed within the observation period. A total of 94 patients had PD at some point during the observation period (regardless of final assessed ORR); of these, 53 patients continued to receive ibrutinib for a duration of ≥ 2 days (median 26 days) after diagnosis of PD.

**Fig. 2:**
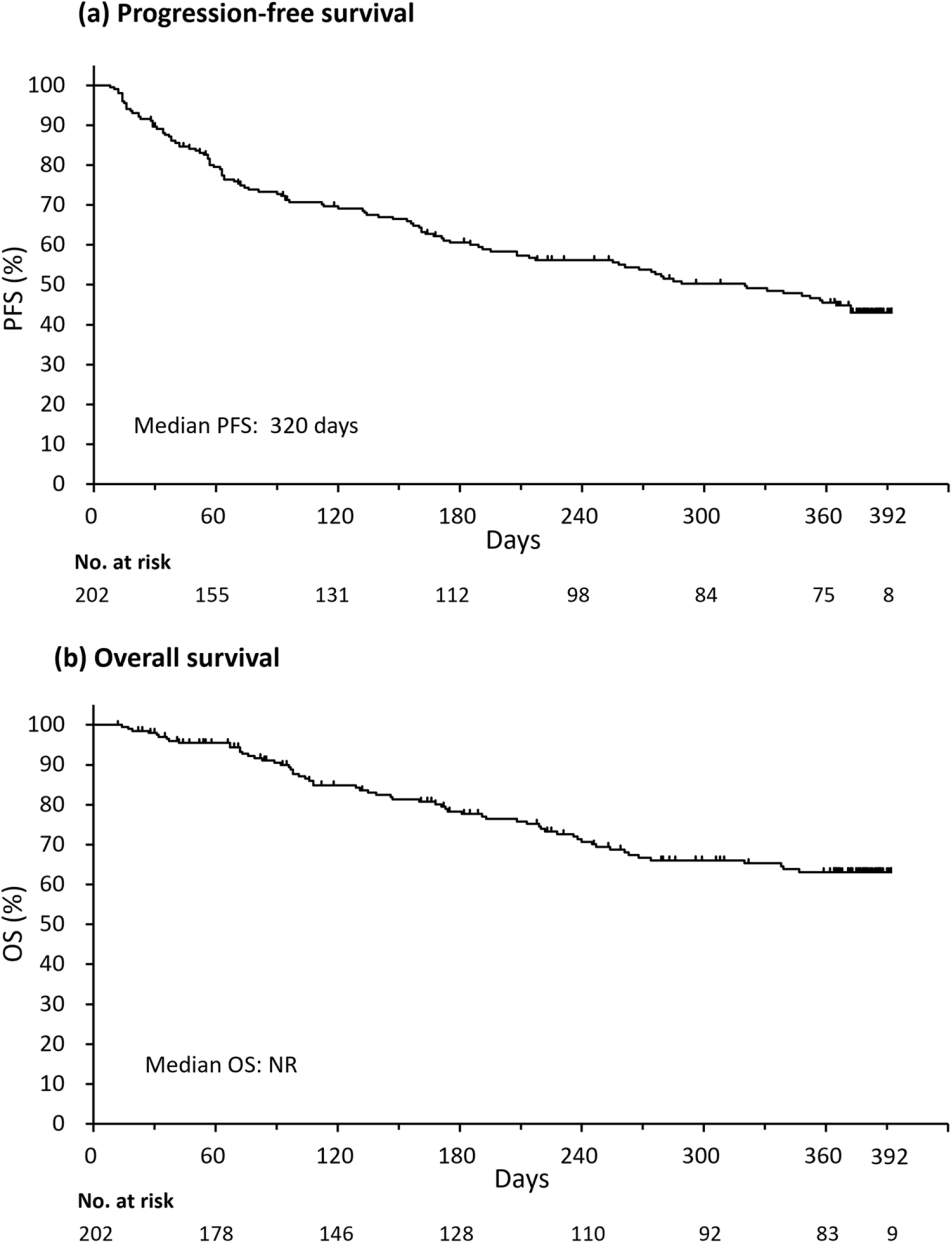
**a** Progression-free survival and **b** overall survival during ibrutinib treatment. Data are for the effectiveness analysis set. *No.* number, *NR* not reached, *OS* overall survival, *PFS* progression-free survival

The 52-week PFS rate was 47.5%, and the 52-week OS rate was 69.3%. Median PFS was 320.0 days (95% confidence interval: 208.0 days, not reached; Fig. 2a) and the median OS had not been reached (Fig. 2b).

### Safety

**Fig. 3:**
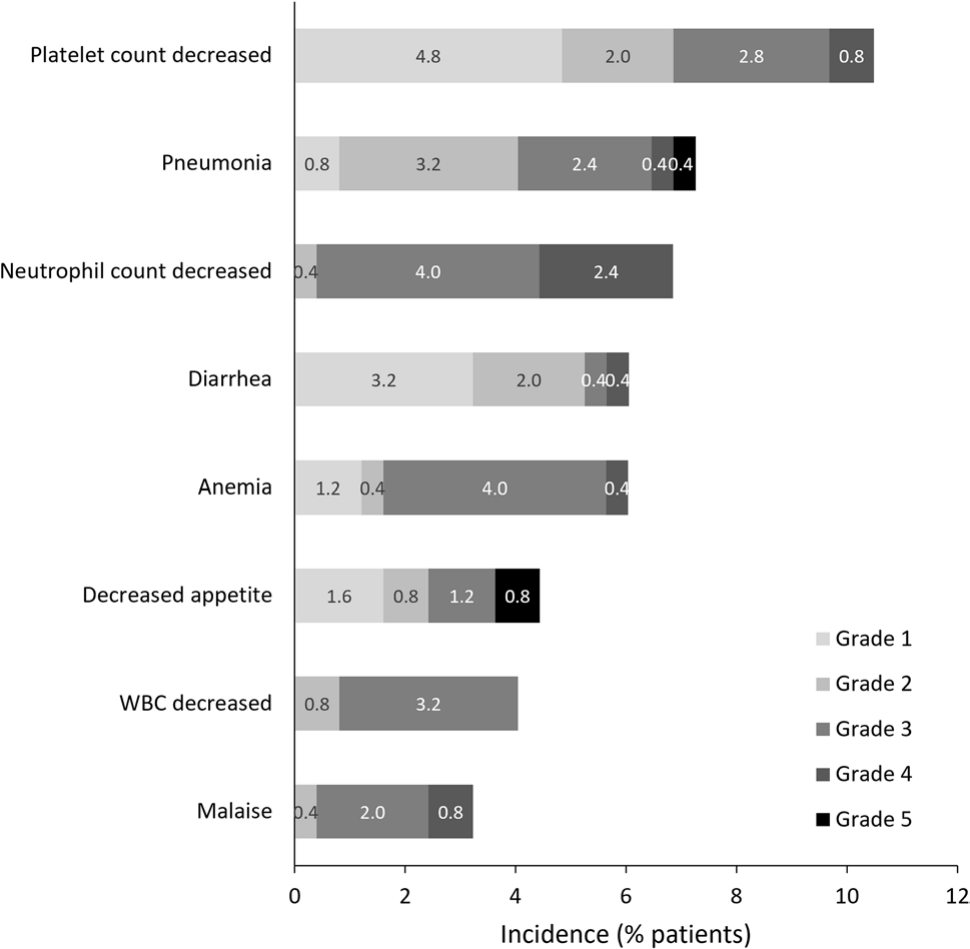
Adverse events (other than mantle cell lymphoma progression and death) occurring in ≥ 3% of patients. *WBC* white blood cell

Overall, 74.6% of patients developed an AE. The most common AEs occurring in ≥ 3.0% of patients were platelet count decreased (occurring in 10.4% of patients), pneumonia (7.2%), neutrophil count decreased (6.8%), diarrhea (6.0%), anemia (6.0%), decreased appetite (4.4%), white blood cell count decreased (4.0%), and malaise (3.2%; Fig. 3).

**Fig. 4:**
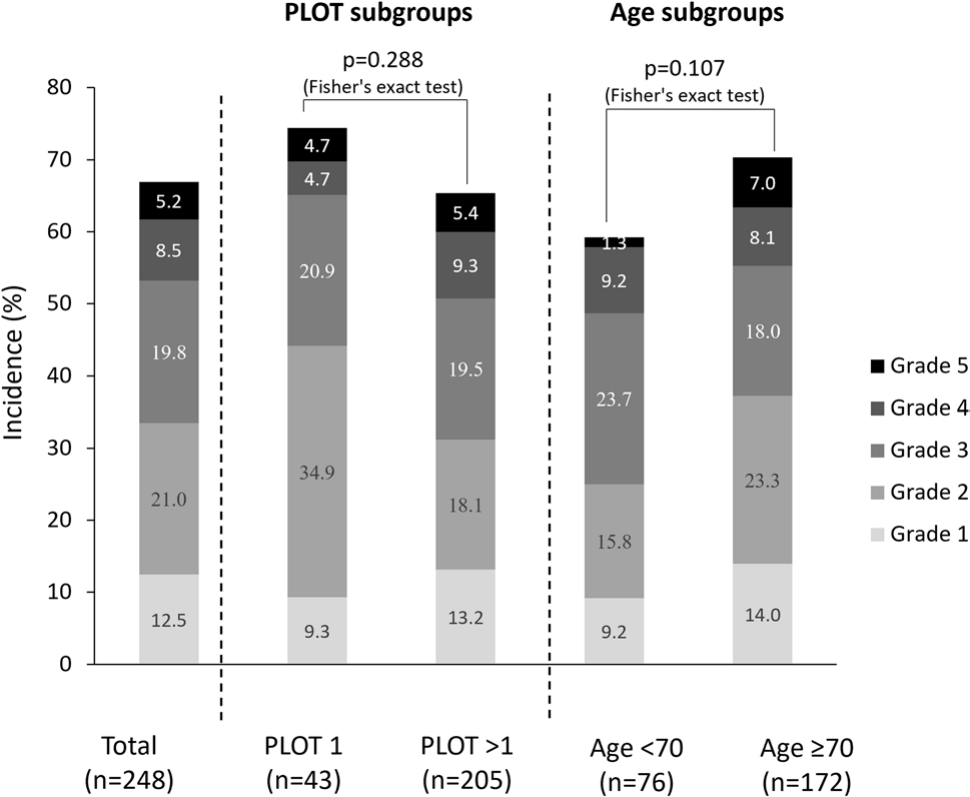
Adverse events (other than mantle cell lymphoma progression and death) by grade, overall and in patient subgroups. *PLOT* previous lines of therapy

Approximately 67.0% of patients developed an AE not related to MCL progression events. Of these AEs, approximately 50.0% were Grade 1 or 2 in severity, and 50.0% were Grade ≥ 3 (Fig. 4). The incidence of these AEs was slightly higher in patients who had received one previous line of therapy than in those who had received > 1 previous line of therapy, and in those aged ≥ 70.0 years than those < 70.0 years (Fig. 4).

#### AEs of special interest

Infections, bleeding, and arrhythmias of any grade occurred in 19.8%, 10.1%, and 2.0% of patients, respectively, and were of Grade ≥ 3 severity in 9.7%, 2.4%, and 0.4% of patients, respectively (Table 3).

**Table 3:** Dose management and outcomes of adverse events of special interest (safety analysis set)

	Infection		Bleeding		Arrhythmia	
	All grades		All grades		All grades	
AE of special interest, *n* (%)	49 (19.8)		25 (10.1)		5 (2.0)	
	Grade 1/2	Grade ≥ 3	Grade 1/2	Grade ≥ 3	Grade 1/2	Grade ≥ 3
AE of special interest, *n* (%)	32 (12.9)	24 (9.7)	19 (7.7)	6 (2.4)	4 (1.6)	1 (0.4)
Ibrutinib dose management, *n* (%)						
No dose change	13 (40.6)	5 (20.8)	6 (31.6)	0	1 (25.0)	0
Dose reduction	5 (15.6)	1 (4.2)	5 (26.3)	0	0	0
Temporary interruption	14 (43.8)	11 (45.8)	6 (31.6)	2 (33.3)	1 (25.0)	0
Discontinuation due to AE	0	7 (29.2)	2 (10.5)	4 (66.7)	2 (50.0)	1 (100.0)
Complete resolution of AEs, *n* (%)	30 (93.8)	18 (75.0)	17 (89.5)	3 (50.0)	2 (50.0)	1 (100.0)
Dose increased after reduction, *n* (%)	4 (80.0)	1 (100.0)	2 (40.0)	0	0	0
Restarted after temporary interruption, *n* (%)	14 (100.0)	8 (72.7)	6 (100.0)	2 (100.0)	1 (100.0)	0
Median (range) no. of AE episodes	1.0 (1.0–2.0)	1.0 (1.0–2.0)	1.0 (1.0–2.0)	1.0 (1.0–2.0)	1.0 (1.0–1.0)	1.0 (1.0–1.0)

*AE* adverse event, *n* number of patients, *no.* number

Infections that occurred in ≥ 5 patients were pneumonia (*n* = 17, 6.9%), bronchitis (*n* = 6, 2.4%), and herpes zoster (*n* = 5, 2.0%; Supplementary Table S2, Electronic Supplementary Materials). Three patients experienced Grade 5 infections that were attributed to ibrutinib treatment. Of these, one patient, who also had Grade 3 anemia, developed bacterial sepsis and pneumonia after approximately 20 weeks of ibrutinib treatment; ibrutinib was immediately discontinued, but the patient died 17 days later. One patient developed meningitis approximately 9 weeks after stopping ibrutinib, having received the drug for approximately 20 weeks prior; they also had Grade 1 pneumonia, Grade 3 herpes zoster oticus, and Grade 3 platelet count decreased. The patient died 5 days after developing meningitis. The third patient developed bacterial pneumonia after approximately 4 weeks of ibrutinib treatment; ibrutinib was discontinued, but the patient died 13 days later.

Overall, 120 (48.3%) patients in the safety analysis set received anti-infective prophylaxis and 30 developed infections (25.0%), compared with 20 infections in the 128 patients who did not receive prophylaxis (15.6%; Supplementary Tables S3 and S4, Electronic Supplementary Materials). The most common prophylaxis was drugs used to treat *Pneumocystis jirovecii* pneumonia (trimethoprim/sulfamethoxazole or atovaquone). Two of the three infection-related deaths occurred in patients who had received anti-infective prophylaxis.

The number of patients requiring dose reduction, interruption, or ibrutinib discontinuation due to AEs of special interest is shown in Table 3. Overall, infections were the cause of permanent treatment discontinuation in seven patients (all Grade ≥ 3), an ibrutinib dose reduction in six (Grade 1 or 2 in five and Grade ≥ 3 in one), and a temporary dose interruption in 25 (Grade 1 or 2 in 14 and Grade ≥ 3 in 11). However, five of the six patients who underwent a dose reduction were able to re-escalate the ibrutinib dose again, and 22 of the 25 for whom a temporary dose interruption was put in place were able to restart ibrutinib after the interruption. Infections completely resolved in 87.8% of patients (43/49 of those with infection) who developed an infectious episode of any grade.

The only bleeding event that occurred in ≥ 5 patients was petechiae (*n* = 5, 2.0%; Supplementary Table S2, Electronic Supplementary Materials). Anticoagulant or antiplatelet agents were being used by 48 patients in the safety analysis set (19.4%), and nine of these patients developed bleeding events (18.8%); the other 16 bleeding events occurred in patients not taking anticoagulant or antiplatelet agents (8.0%). The type and doses of anticoagulant/antiplatelet therapies are shown in Supplementary Table S5 in the Electronic Supplementary Materials. Five of the 16 patients taking aspirin and four of the six patients taking a direct-acting oral anticoagulant (DOAC) developed bleeding during ibrutinib treatment.

Bleeding led to permanent ibrutinib discontinuation in six patients (two with Grade 1 or 2 and four with Grade ≥ 3 bleeding), an ibrutinib dose reduction in five (all Grade 1 or 2), and a temporary dose interruption in eight (six with Grade 1 or 2 and two with Grade ≥ 3 bleeding; Table 3), but two of the five patients were able to increase the ibrutinib dose again, and all eight were able to restart ibrutinib after the temporary interruption. Bleeding completely resolved in 80.0% of patients who developed a bleeding episode of any grade.

The five patients who experienced arrhythmias all developed atrial fibrillation (AF; Supplementary Table S2, Electronic Supplementary Materials); AF developed in two of 13 patients who had concurrent arrhythmia at baseline (15.4%) and in three of 230 patients with no concurrent arrhythmias (1.3%; Supplementary Table S6, Electronic Supplementary Materials). No patients with a past medical history of arrhythmia developed AF during ibrutinib treatment. Three patients who developed AF (two with Grade 1 or 2 AF and one with Grade ≥ 3 AF) permanently discontinued ibrutinib (Table 3). Another patient who developed AF had a temporary dose interruption, but was able to restart ibrutinib again once the AE had resolved. The remaining patient who developed AF did not require a dose change. AF completely resolved in three patients (60.0%) who developed AF of any grade.

**Fig. 5:**
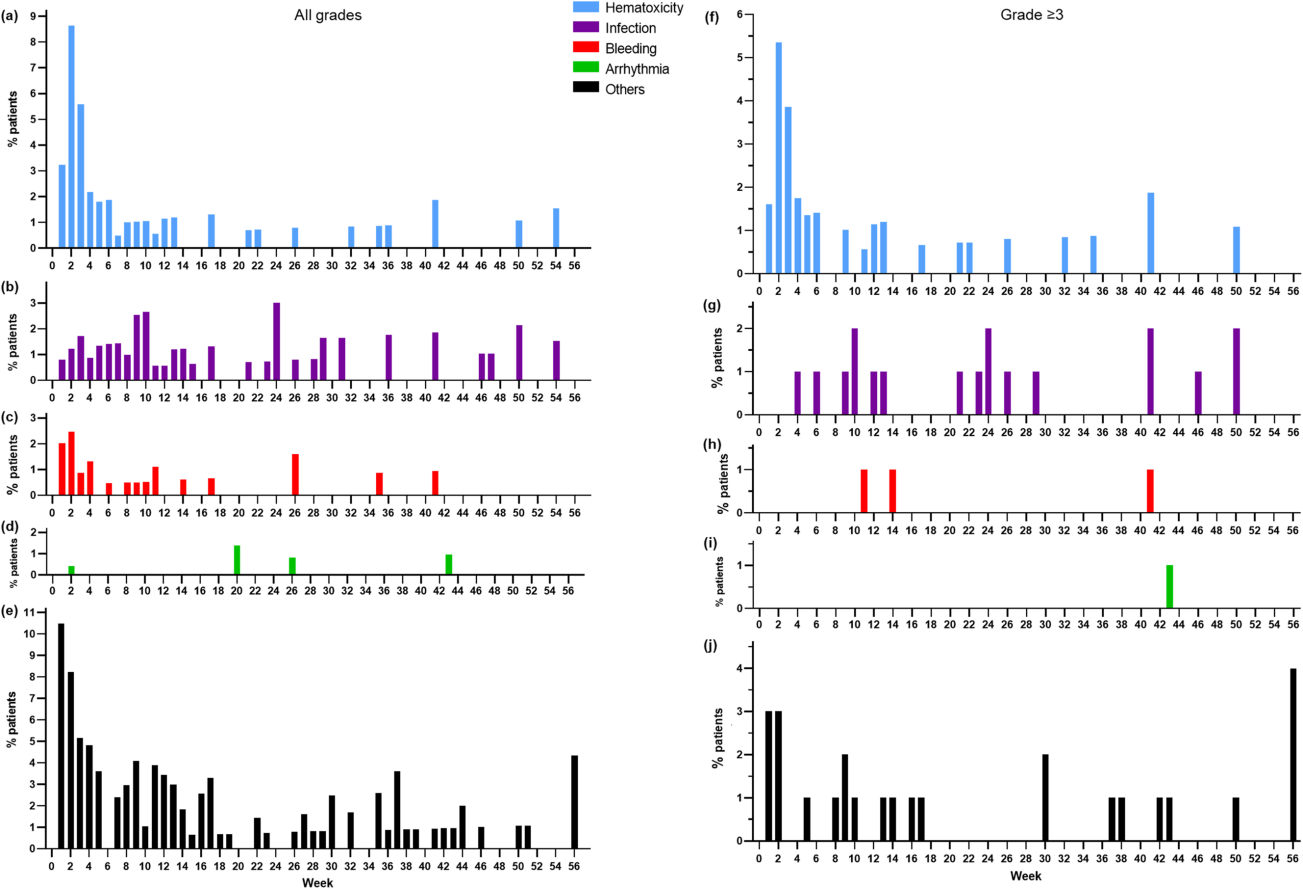
Timing of onset for adverse events of special interest of **a**–**e** all grades and **f**–**j** Grade ≥ 3 in the safety analysis set (*N* = 248)

The incidence of AEs of special interest, including Grade ≥ 3 AEs, was higher during the first 10 weeks after ibrutinib initiation, and decreased thereafter (Fig. 5).

## Discussion

This all-case PMS demonstrated the real-world effectiveness and safety of ibrutinib in Japanese patients with R/R MCL, confirming that the effectiveness/safety is consistent with the known profile on approval in Japan. No new safety concerns were identified.

The ORR in our PMS (59.9%) was lower than that reported in the Japanese phase II study (87.5%) [10, 11] and in the international phase II and phase III studies in patients with R/R MCL (63.0–72.0%) [12, 14, 15, 18]. This may be due to a higher grade of target disease in the PMS and differences between studies in the dose of ibrutinib administered. However, the 52-week OS rate (69.3%), 52-week PFS rate (47.5%), and median PFS (320.0 days; approximately 11 months) in our PMS was comparable with those reported in the phase II and III studies (1 year OS rate, 68.0% [12]; 1 year PFS rate, 47.0% [15]; median PFS, 10.5–14.6 months [12, 14, 15]).

The overall incidence of AEs in the current PMS (74.6%) was very similar to the incidence in a PMS in patients with R/R CLL/SLL (74.0%) [17], but lower than in the Japanese phase II study (100.0%) [10, 11], and the international phase III study of patients with R/R MCL [12]. Similarly, the incidence of specific AEs, such as platelet count decreased (10.5% vs 31.3%), diarrhea (6.1% vs 37.5%), anemia (6.0% vs 25.0%), decreased appetite (4.4% vs 18.8%), and malaise (3.2% vs 12.5%), was lower in the current PMS than in the previous phase II study [10, 11]. There may be several reasons for the lower rate of AEs in our PMS than in the previous phase II trial. First, active assessment of AEs is generally more rigorous in a clinical trial setting than in clinical practice. Second, dose intensity is often lower in clinical practice than in clinical trials. Third, a high proportion of patients in the current PMS received prophylactic treatments to prevent AEs (e.g., 48.0% of patients in our PMS received anti-infective prophylaxis), whereas the phase II or III trials did not routinely administer such therapies [10–12].

Importantly, the incidence of specific AEs in our population of R/R MCL patients was highly comparable with their incidence in a previous real-world population of R/R CLL/SLL patients [17]. In both PMSs AEs of special interest, including infections, bleeding, and arrhythmia, occurred infrequently, and were manageable with dose modifications. Moreover, AEs were not statistically significantly more frequent in patients aged > 70.0 years than in younger patients, or in patients who had received more than one previous line of therapy compared with only one previous line.

AF developed in 2.0% of patients, which is lower than in the phase III randomized study in patients with R/R MCL (4.0%) [12], or in a pooled analysis of data from ibrutinib clinical trials (4.6%) [18]. In our PMS, the incidence of AF was significantly higher in patients with a concurrent arrhythmia diagnosis at baseline, but not in those with a past history of arrhythmias. In this respect, our data differ from the PMS in patients with R/R CLL/SLL [17] and a retrospective chart review [19], which showed, respectively, that a past history of arrhythmias and heart failure increased the risk of AF development during ibrutinib. Other studies have shown that structural heart disease is a risk factor for AF development during ibrutinib [20]. While further study is necessary, the results of this PMS and previous studies suggest that careful assessment of a patient’s cardiovascular history and status should be considered prior to initiating ibrutinib.

Bleeding events developed in 25 patients (10.1%), nine of whom were receiving antiplatelet or anticoagulant therapy. Five of the 16 patients taking aspirin and four of the six taking DOACs developed bleeding. This is consistent with data from an integrated analysis of bleeding in ibrutinib clinical trials, which showed that antiplatelet and anticoagulant treatment increased the risk of major bleeding [21]. Anemia is another independent risk factor for bleeding during ibrutinib treatment [21].

Encouragingly, a high proportion of patients with AEs of special interest (including > 50.0% of those who experienced a Grade ≥ 3 AE) had complete resolution of their AE, and the majority of patients in whom a dose reduction/temporary interruption was instituted were able to subsequently resume ibrutinib treatment or resume the previous ibrutinib dose.

Our PMS has a number of strengths. Firstly, despite the rarity of R/R MCL, we were able to enroll a large number of patients. Secondly, because our PMS enrolled all patients who started ibrutinib, it is highly representative of the R/R MCL patient population in clinical practice in Japan. Indeed, the age and sex of the patients in our PMS were very comparable with data from the CLIMBER-DBR database study of patients with MCL in Japan, which examined clinical characteristics, treatment patterns and healthcare resource utilization in this patient population [4].

The limitations of the PMS are those inherent to observational research, including: the lack of an independent control arm; a relatively short (52-week) observation period; response being evaluated at the physicians’ discretion rather than using a pre-defined schedule; AEs being potentially under-reported; the reporting of PD or death due to MCL as AEs may make interpretation of ibrutinib discontinuation rates difficult; and incomplete or missing data for some patients. For example, data for t(11; 14) translocation and cyclin D1 expression were missing for 53–102 patients, which limits certainty that patients had a definitive diagnosis of MCL; however, it is possible that such testing (and thus a definitive diagnosis of MCL made) had been conducted at a treatment facility different to the facility at which patients were enrolled into this PMS. Standard treatment effectiveness endpoints were evaluated, including ORR, PFS and OS; however, future studies of ibrutinib should evaluate the time to next treatment to determine the extent of use of ibrutinib based on patient treatment response in a real-world clinical practice setting.

In conclusion, these results suggest that ibrutinib has sustained effectiveness and acceptable tolerability in Japanese patients with R/R MCL in routine clinical practice. No unexpected AEs were identified, and AEs that did occur were mostly manageable and resolved after treatment interruption or dose reduction.

### Supplementary Information

Below is the link to the electronic supplementary material.

Supplementary file1 (DOCX 26 KB)

## Data Availability

The datasets generated during and/or analyzed during the current study are not publicly available due to confidentiality clauses signed with the participating medical institutions.
