# Supplementary material for: Real-world effectiveness and safety of ibrutinib in relapsed/refractory mantle cell lymphoma in Japan: post-marketing surveillance
Source: Int J Hematol. 2024 Jan 9;119(2):146–55. doi: 10.1007/s12185-023-03687-8 (PMC10830708; doi:10.1007/s12185-023-03687-8)
Supplement: Supplementary file 1 — Supplementary file1 (DOCX 26 KB) [file 12185_2023_3687_MOESM1_ESM.docx]

**The Real-World Effectiveness and Safety of Ibrutinib in Relapsed/Refractory Mantle Cell Lymphoma in Japan: A Post-Marketing Surveillance**

**Electronic Supplementary Material**

**Authors:** Dai Maruyama, Ai Omi, Fumi Nomura, Tokiko Touma, Yukiko Noguchi, Kyoko Takebe, Koji Izutsu

**Corresponding author:** Ai Omi

**E-mail:** [AOmi@ITS.JNJ.com](mailto:AOmi@ITS.JNJ.com)

**Supplementary Table S1** Prior therapies (safety analysis set)

| Prior therapies, *n* (%) | Safety analysis set (*N*=248) |
| --- | --- |
| *Transplantation* | 44 (17.7) |
| *Drug therapy* | 248 (100.0) |
| Rituximab + bendamustine | 171 (69.0) |
| R-CHOP | 129 (52.0) |
| Rituximab | 110 (44.4) |
| VR-CAP | 39 (15.7) |
| Hyper-CVAD | 32 (12.9) |
| Bendamustine | 28 (11.3) |
| Methotrexate + cytarabine | 26 (10.5) |
| Fludarabine | 21 (8.5) |
| R-CVP | 4 (1.6) |
| Other | 148 (59.7) |

Hyper-CVAD, hyperfractionated cyclophosphamide + vincristine + doxorubicin (Adriamycin) + dexamethasone; n, number of patients; R-CHOP, rituximab + cyclophosphamide + doxorubicin + vincristine (Oncovin) + prednisone; R-CVP, rituximab + cyclophosphamide + vincristine + prednisone; VR-CAP, bortezomib (Velcade) + rituximab + cyclophosphamide + doxorubicin (Adriamycin) + prednisone

**Supplementary Table S2** Incidence of adverse events of special interest in the safety analysis set (N=248)

| AEs of special interest, *n* (%) | | | | | |
| --- | --- | --- | --- | --- | --- |
| Infections | | Bleeding | | Arrhythmias | |
| *Any infection* | 49 (19.8) | *Any bleeding* | 25 (10.1) | *Any arrhythmia* | 5 (2.0) |
| *Specific infections in ≥2 patients* |  | *Specific events in ≥2 patients* |  | *Specific events in ≥2 patients* |  |
| Pneumonia | 17 (6.9) | Petechiae | 5 (2.0) | Atrial fibrillation | 5 (2.0) |
| Bronchitis | 6 (2.4) | Epistaxis | 4 (1.6) |  |  |
| Herpes zoster | 5 (2.0) | Hematuria | 3 (1.2) |  |  |
| Pneumonia bacterial | 4 (1.6) | Subdural hematoma | 2 (0.8) |  |  |
| Cystitis | 3 (1.2) | Hemoptysis | 2 (0.8) |  |  |
| Infection | 2 (0.8) | Hemorrhage subcutaneous | 2 (0.8) |  |  |
| Nasopharyngitis | 2 (0.8) |  |  |  |  |
| Oral candidiasis | 2 (0.8) |  |  |  |  |

AE, adverse event; n, number of patients

**Supplementary Table S3** Incidence of infections and infestations in patients who had or had not received anti-infective prophylaxis

| *n* (%) | With prophylaxis (*n*=120) | Without prophylaxis (*n*=128) |
| --- | --- | --- |
| *Any infection or infestation* | 30 (25.0) | 20 (15.6) |
| *Severity of infection or infestation* |  |  |
| Grade 1–2 | 21 (17.5) | 11 (8.6) |
| Grade 3–4 | 13 (10.8) | 9 (7.0) |
| Grade 5 | 2 (1.7) | 1 (<0.1) |
| *Type of infections or infestations* | 30 (25.0) | 20 (15.6) |
| Appendicitis | 0 | 1 (0.8) |
| Bronchitis | 3 (2.5) | 3 (2.3) |
| Bronchopulmonary aspergillosis | 0 | 1 (0.8) |
| Cellulitis | 0 | 1 (0.8) |
| Chronic sinusitis | 1 (0.8) | 0 |
| Cystitis | 3 (2.5) | 0 |
| Furuncle | 1 (0.8) | 0 |
| Gastroenteritis | 1 (0.8) | 0 |
| Herpes zoster | 1 (0.8) | 4 (3.1) |
| Infection | 1 (0.8) | 1 (0.8) |
| Meningitis | 1 (0.8) | 0 |
| Nasopharyngitis | 1 (0.8) | 1 (0.8) |
| Oral candidiasis | 1 (0.8) | 1 (0.8) |
| Otitis media | 0 | 1 (0.8) |
| Paronychia | 1 (0.8) | 0 |
| Pharyngitis | 1 (0.8) | 0 |
| Pneumonia | 12 (10.0) | 5 (3.9) |
| Pneumonia pneumococcal | 0 | 1 (0.8) |
| Sepsis | 0 | 1 (0.8) |
| Skin infection | 1 (0.8) | 0 |
| Urinary tract infection | 1 (0.8) | 0 |
| Anal abscess | 1 (0.8) | 0 |
| Cytomegalovirus enterocolitis | 1 (0.8) | 0 |
| Staphylococcal bacteremia | 1 (0.8) | 0 |
| Bacterial sepsis | 0 | 1 (0.8) |
| Enteritis infectious | 1 (0.8) | 0 |
| Pneumonia bacterial | 4 (3.3) | 0 |
| Respiratory tract infection | 0 | 1 (0.8) |
| Herpes zoster oticus | 1 (0.8) | 0 |
| Lymphadenitis bacterial | 0 | 1 (0.8) |
| *Pneumocystis jirovecii* pneumonia | 0 | 1 (0.8) |

n, number of patients; PJP, *Pneumocystis jirovecii* pneumonia

**Supplementary Table S4** Use of anti-infective prophylaxes (safety analysis set)

| *n* (%) | Safety analysis set (*N*=248) |
| --- | --- |
| Any prophylaxis | 120 (48.4) |
| *Type of prophylaxis* |  |
| Antibacterial drug | 5 (2.0) |
| Antiviral drug | 72 (29.0) |
| Antifungal drug | 23 (9.3) |
| Anti-PJP drug^a^ | 94 (37.9) |

^a^Trimethoprim/sulfamethoxazole (n=91) + atovaquone (n=3)

**Supplementary Table S5** Use of anticoagulant and antiplatelet medication, and relationship to bleeding events

|  | *n* | Median no. of dosing days during follow-up | No. of patients with bleeding events | | Median age, years | Median initial anticoagulant dose, mg |
| --- | --- | --- | --- | --- | --- | --- |
|  |  |  | Causal relationship to ibrutinib | No causal relationship to ibrutinib |  |  |
| *Any antiplatelet or anticoagulant* | 48 | 75.5 | 7 | 2 | – | – |
| Aspirin | 16 | 157.0 | 3 | 2 | – | – |
| Heparin | 4 | 115.0 | 0 | 0 | – | – |
| Vitamin K antagonist | 4 | 84.0 | 0 | 0 | – | – |
| *DOAC* | 6 | 59.0 | 4 | 0 | – | – |
| Apixaban | 2 | 186.5 | 1 | 0 | 76.0 | 6.0 |
| Rivaroxaban | 3 | 46.0 | 2 | 0 | 78.0 | 10.0 |
| Edoxaban | 1 | 74.0 | 1 | 0 | 79.0 | 60.0 |
| *Other* | 25 | 15.0 | 1 | 0 | – | – |

DOAC, direct-acting oral anticoagulant; N, number of patients; no., number

**Supplementary Table S6** Number of patients developing arrhythmia as a proportion of those with a past medical history of arrhythmias or concurrent diagnosis of arrhythmia

|  | *n* | Patients with arrhythmias *n* (%) | P-value* |
| --- | --- | --- | --- |
| *Past medical history of arrhythmias* |  |  |  |
| Yes | 4 | 0 | – |
| No | 239 | 5 (2.1) |  |
| *Concurrent arrhythmia at baseline* |  |  |  |
| Yes | 13 | 2 (15.4) | 0.01 |
| No | 230 | 3 (1.3) |  |

*Fisher’s exact test

N/n, number of patients
